# (Methanol-κ*O*){1-[2-(piperazin-4-ium-1-yl-κ*N* ^1^)ethyl­imino­methyl-κ*N*]naphthalen-2-olato-κ*O*}bis­(thio­cyanato-κ*N*)nickel(II) methanol monosolvate

**DOI:** 10.1107/S1600536812013773

**Published:** 2012-04-04

**Authors:** Pin-Ai Li

**Affiliations:** a Luohe Medical College, Luohe Henan 462002, People’s Republic of China

## Abstract

In the title solvated complex, [Ni(C_17_H_21_N_3_O)(NCS)_2_(CH_3_OH)]·CH_3_OH, the Ni^2+^ ion is coordinated by one phenolate O, one imine N, and one amine N atom of the tridentate Schiff base ligand, two thio­cyanate N atoms and one methanol O atom, resulting in a distorted *cis*-NiO_2_N_4_ octa­hedral geometry. The chelate ring formed by the phenolate O and imine N atoms approximates to an envelope with the Ni atom as the flap, whereas the chelate ring formed by the two N atoms is twisted about the C—C bond. In the crystal, the components are linked by O—H⋯O, N—H⋯O, N—H⋯S, and O—H⋯S hydrogen bonds.

## Related literature

For background to the biological properties of nickel complexes of Schiff bases, see: Chohan & Kausar (1993 ▶); Osowole *et al.* (2008 ▶); Arif *et al.* (2011 ▶). For related structures, see: Ji & Lu (2010 ▶); Wang (2010 ▶); Xue *et al.* (2010 ▶). 
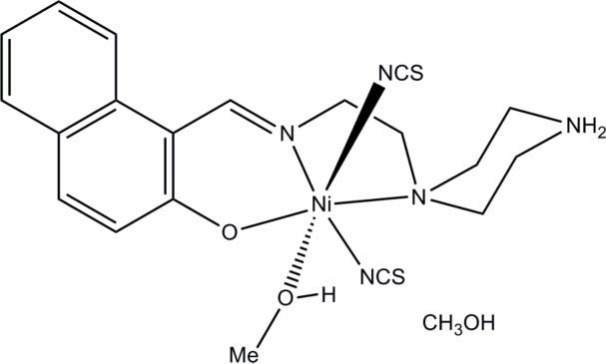


## Experimental

### 

#### Crystal data

[Ni(C_17_H_21_N_3_O)(NCS)_2_(CH_4_O)]·CH_4_O
*M* *_r_* = 522.32
Monoclinic, 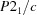

*a* = 9.7420 (19) Å
*b* = 15.304 (3) Å
*c* = 18.302 (5) Å
β = 116.01 (2)°
*V* = 2452.3 (10) Å^3^
*Z* = 4
Mo *K*α radiation
μ = 0.99 mm^−1^
*T* = 298 K
0.17 × 0.15 × 0.15 mm

#### Data collection

Bruker SMART 1000 CCD diffractometer
Absorption correction: multi-scan (*SADABS*; Sheldrick, 2000 ▶) *T*_min_ = 0.849, *T*_max_ = 0.865
16581 measured reflections
4196 independent reflections
2354 reflections with *I* > 2σ(*I*)
*R*_int_ = 0.153

#### Refinement

*R*[*F* ^2^ > 2σ(*F* ^2^)] = 0.092
*wR*(*F* ^2^) = 0.209
*S* = 1.00
4196 reflections
295 parameters
1 restraint
H atoms treated by a mixture of independent and constrained refinement
Δρ_max_ = 1.05 e Å^−3^
Δρ_min_ = −0.47 e Å^−3^

### 

Data collection: *SMART* (Bruker, 2000 ▶); cell refinement: *SAINT* (Bruker, 2000 ▶); data reduction: *SAINT*; program(s) used to solve structure: *SHELXTL* (Sheldrick, 2008 ▶); program(s) used to refine structure: *SHELXTL*; molecular graphics: *SHELXTL*; software used to prepare material for publication: *SHELXTL*.

## Supplementary Material

Crystal structure: contains datablock(s) I, global. DOI: 10.1107/S1600536812013773/hb6710sup1.cif

Structure factors: contains datablock(s) I. DOI: 10.1107/S1600536812013773/hb6710Isup2.hkl

Additional supplementary materials:  crystallographic information; 3D view; checkCIF report

## Figures and Tables

**Table 1:** Selected bond lengths (Å)

Ni1—N1	1.997 (6)
Ni1—N3	2.044 (7)
Ni1—O1	2.049 (5)
Ni1—N4	2.064 (7)
Ni1—O2	2.128 (7)
Ni1—N2	2.241 (6)

**Table 2:** Hydrogen-bond geometry (Å, °)

*D*—H⋯*A*	*D*—H	H⋯*A*	*D*⋯*A*	*D*—H⋯*A*
O2—H2⋯O3	0.82 (1)	2.03 (4)	2.793 (10)	155 (10)
N5—H5*B*⋯O1^i^	0.90	1.75	2.649 (8)	175
N5—H5*A*⋯S2^ii^	0.90	2.67	3.480 (7)	150
O3—H3⋯S2^ii^	0.82	2.78	3.532 (9)	154

Symmetry codes: (i) 
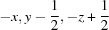
; (ii) 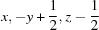.

**Fig. 1.**
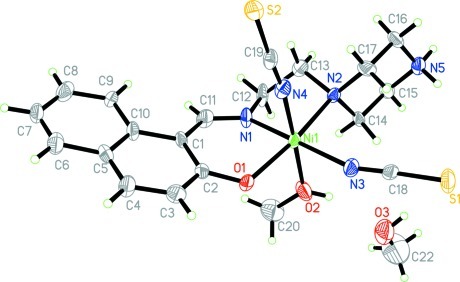
The structure of the title compound, with displacement ellipsoids drawn at the 30% probability level for Non-H atoms.

**Fig. 2.**
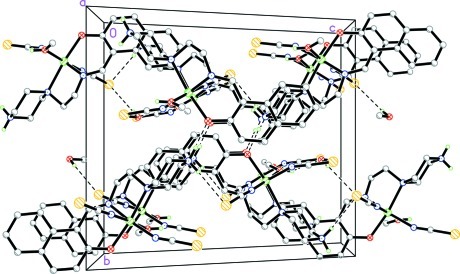
The crystal structure of the title compound, viewed along the a axis. Hydrogen bonds are shown as dashed lines.

## supplementary crystallographic information

### Comment

Soem nickel complexes derived from Schiff bases have possess interesting biological properties (Arif *et al.*, 2011; Osowole *et al.*, 2008; Chohan & Kausar, 1993). As an extension of the work on the structures of such complexes, the author reports herein the title new nickel complex.

The title compound contains a mononuclear nickel complex molecule and a methanol molecule of crystallization (Fig. 1). The Ni atom in the complex is coordinated by one phenolate O, one imine N, and one amine N atom of the Schiff base ligand, two thiocyanate N atoms, and one methanol O atom, forming an octahedral coordination. The bond lengths (Table 1) are comparable to those reported in the similar nickel complexes with Schiff bases (Wang, 2010; Ji & Lu, 2010; Xue *et al.*, 2010). The crystal structure features N—H···O, N—H···S, and O—H···S hydrogen bonds (Table 2, Fig. 2).

### Experimental

2-Hydroxy-1-naphthaldehyde (1.72 g, 0.01 mol) and 2-piperazin-1-ylethyamine (1.29 g, 0.01 mol) were mixed in methanol (30 ml). To the stirred mixture was added a methanolic solution (10 ml) of ammonium thiocyanate (1.52 g, 0.02 mol) and a methanolic solution (10 ml) of nickel nitrate (2.91 g, 0.01 mol). The final mixture was further stirred for 30 min to give a green solution. Green block-like single crystals were obtained by slow evaporation of the solution in air.

### Refinement

H2 was located from a difference Fourier map and refined isotropically, with O—H distance restrained to 0.82 (1) Å. The remaining hydrogen atoms were placed in calculated positions, with C—H distances in the range 0.93–0.97 Å, O—H distance of 0.82 Å, and with *U*_iso_ values set to 1.2*U*_eq_(C) and 1.5*U*_eq_(methyl C and O3).

### Crystal data

**Table:**

[Ni(C_17_H_21_N_3_O)(NCS)_2_(CH_4_O)]·CH_4_O	*F*(000) = 1096
*M**_r_* = 522.32	*D*_x_ = 1.415 Mg m^−^^3^
Monoclinic, *P*2_1_/*c*	Mo *K*α radiation, λ = 0.71073 Å
*a* = 9.7420 (19) Å	Cell parameters from 888 reflections
*b* = 15.304 (3) Å	θ = 2.3–24.5°
*c* = 18.302 (5) Å	µ = 0.99 mm^−^^1^
β = 116.01 (2)°	*T* = 298 K
*V* = 2452.3 (10) Å^3^	Block, green
*Z* = 4	0.17 × 0.15 × 0.15 mm

### Data collection

**Table:**

Bruker SMART 1000 CCD diffractometer	4196 independent reflections
Radiation source: fine-focus sealed tube	2354 reflections with *I* > 2σ(*I*)
Graphite monochromator	*R*_int_ = 0.153
ω scan	θ_max_ = 25.2°, θ_min_ = 1.8°
Absorption correction: multi-scan (*SADABS*; Sheldrick, 2000)	*h* = −11→11
*T*_min_ = 0.849, *T*_max_ = 0.865	*k* = −18→18
16581 measured reflections	*l* = −21→21

### Refinement

**Table:**

Refinement on *F*^2^	Primary atom site location: structure-invariant direct methods
Least-squares matrix: full	Secondary atom site location: difference Fourier map
*R*[*F*^2^ > 2σ(*F*^2^)] = 0.092	Hydrogen site location: inferred from neighbouring sites
*wR*(*F*^2^) = 0.209	H atoms treated by a mixture of independent and constrained refinement
*S* = 1.00	*w* = 1/[σ^2^(*F*_o_^2^) + (0.0864*P*)^2^] where *P* = (*F*_o_^2^ + 2*F*_c_^2^)/3
4196 reflections	(Δ/σ)_max_ < 0.001
295 parameters	Δρ_max_ = 1.05 e Å^−^^3^
1 restraint	Δρ_min_ = −0.47 e Å^−^^3^

### Special details

**Table:**

Geometry. All e.s.d.'s (except the e.s.d. in the dihedral angle between two l.s. planes) are estimated using the full covariance matrix. The cell e.s.d.'s are taken into account individually in the estimation of e.s.d.'s in distances, angles and torsion angles; correlations between e.s.d.'s in cell parameters are only used when they are defined by crystal symmetry. An approximate (isotropic) treatment of cell e.s.d.'s is used for estimating e.s.d.'s involving l.s. planes.
Refinement. Refinement of *F*^2^ against ALL reflections. The weighted *R*-factor *wR* and goodness of fit *S* are based on *F*^2^, conventional *R*-factors *R* are based on *F*, with *F* set to zero for negative *F*^2^. The threshold expression of *F*^2^ > σ(*F*^2^) is used only for calculating *R*-factors(gt) *etc*. and is not relevant to the choice of reflections for refinement. *R*-factors based on *F*^2^ are statistically about twice as large as those based on *F*, and *R*- factors based on ALL data will be even larger.

### Fractional atomic coordinates and isotropic or equivalent isotropic displacement parameters (Å^2^)

**Table:**

	*x*	*y*	*z*	*U*_iso_*/*U*_eq_	
Ni1	0.08725 (12)	0.32345 (6)	0.35614 (6)	0.0356 (3)	
S1	−0.3385 (3)	0.40557 (18)	0.11040 (14)	0.0662 (8)	
S2	−0.1939 (3)	0.23841 (17)	0.50772 (14)	0.0641 (8)	
O1	0.1635 (7)	0.4331 (3)	0.4268 (3)	0.0465 (15)	
O2	0.2267 (8)	0.3634 (4)	0.2996 (4)	0.0612 (17)	
O3	0.0875 (10)	0.4095 (6)	0.1353 (4)	0.097 (3)	
H3	0.0055	0.3840	0.1128	0.145*	
N1	0.2670 (7)	0.2604 (4)	0.4397 (4)	0.0437 (18)	
N2	0.0484 (7)	0.1938 (4)	0.2933 (4)	0.0348 (15)	
N3	−0.0853 (8)	0.3835 (4)	0.2599 (4)	0.0488 (19)	
N4	−0.0474 (8)	0.2903 (4)	0.4131 (4)	0.0412 (17)	
N5	−0.1175 (7)	0.0886 (4)	0.1435 (4)	0.0450 (18)	
H5A	−0.1729	0.1302	0.1080	0.054*	
H5B	−0.1389	0.0370	0.1173	0.054*	
C1	0.3224 (8)	0.3598 (5)	0.5527 (4)	0.0374 (19)	
C2	0.2355 (9)	0.4313 (5)	0.5085 (5)	0.041 (2)	
C3	0.2202 (10)	0.5057 (6)	0.5479 (5)	0.053 (2)	
H3A	0.1582	0.5512	0.5174	0.063*	
C4	0.2957 (11)	0.5132 (6)	0.6318 (5)	0.054 (2)	
H4	0.2873	0.5643	0.6569	0.065*	
C5	0.3847 (9)	0.4438 (5)	0.6788 (5)	0.043 (2)	
C6	0.4632 (11)	0.4468 (7)	0.7668 (5)	0.058 (3)	
H6	0.4564	0.4978	0.7926	0.070*	
C7	0.5449 (10)	0.3806 (7)	0.8133 (5)	0.054 (3)	
H7	0.5940	0.3861	0.8696	0.065*	
C8	0.5545 (10)	0.3047 (7)	0.7763 (5)	0.060 (3)	
H8	0.6101	0.2581	0.8082	0.072*	
C9	0.4832 (9)	0.2953 (6)	0.6919 (5)	0.046 (2)	
H9	0.4903	0.2426	0.6685	0.055*	
C10	0.3988 (9)	0.3667 (6)	0.6409 (4)	0.042 (2)	
C11	0.3444 (11)	0.2811 (5)	0.5145 (5)	0.052 (2)	
H11	0.4209	0.2428	0.5471	0.062*	
C12	0.2999 (10)	0.1768 (6)	0.4112 (5)	0.055 (3)	
H12A	0.3612	0.1398	0.4570	0.066*	
H12B	0.3554	0.1865	0.3790	0.066*	
C13	0.1465 (10)	0.1340 (5)	0.3598 (5)	0.049 (2)	
H13A	0.1623	0.0800	0.3367	0.059*	
H13B	0.0960	0.1199	0.3937	0.059*	
C14	0.0892 (9)	0.1941 (5)	0.2235 (4)	0.040 (2)	
H14A	0.1981	0.2043	0.2441	0.048*	
H14B	0.0366	0.2421	0.1875	0.048*	
C15	0.0481 (10)	0.1082 (5)	0.1741 (5)	0.043 (2)	
H15A	0.0732	0.1135	0.1285	0.052*	
H15B	0.1076	0.0606	0.2082	0.052*	
C16	−0.1588 (10)	0.0854 (5)	0.2119 (5)	0.048 (2)	
H16A	−0.1049	0.0375	0.2478	0.058*	
H16B	−0.2675	0.0745	0.1912	0.058*	
C17	−0.1185 (10)	0.1709 (5)	0.2595 (5)	0.048 (2)	
H17A	−0.1780	0.2177	0.2242	0.057*	
H17B	−0.1467	0.1665	0.3040	0.057*	
C18	−0.1908 (10)	0.3914 (5)	0.1988 (5)	0.0382 (19)	
C19	−0.1038 (10)	0.2711 (5)	0.4529 (5)	0.041 (2)	
C20	0.3836 (12)	0.3845 (8)	0.3386 (7)	0.087 (4)	
H20A	0.3976	0.4441	0.3269	0.130*	
H20B	0.4389	0.3466	0.3192	0.130*	
H20C	0.4208	0.3773	0.3962	0.130*	
C22	0.1608 (19)	0.4057 (12)	0.0882 (9)	0.148 (7)	
H22A	0.1522	0.4610	0.0618	0.222*	
H22B	0.1160	0.3609	0.0480	0.222*	
H22C	0.2666	0.3925	0.1212	0.222*	
H2	0.189 (11)	0.361 (7)	0.2499 (8)	0.080*	

### Atomic displacement parameters (Å^2^)

**Table:**

	*U*^11^	*U*^22^	*U*^33^	*U*^12^	*U*^13^	*U*^23^
Ni1	0.0411 (6)	0.0276 (5)	0.0256 (5)	0.0056 (5)	0.0031 (4)	0.0015 (4)
S1	0.0644 (17)	0.0600 (17)	0.0413 (14)	0.0051 (13)	−0.0072 (12)	−0.0074 (12)
S2	0.084 (2)	0.0604 (17)	0.0447 (14)	−0.0249 (14)	0.0252 (14)	−0.0107 (12)
O1	0.077 (4)	0.017 (3)	0.028 (3)	0.012 (3)	0.006 (3)	0.002 (2)
O2	0.062 (5)	0.062 (4)	0.051 (4)	−0.018 (3)	0.017 (4)	−0.021 (4)
O3	0.100 (7)	0.124 (8)	0.056 (5)	−0.019 (6)	0.025 (5)	−0.004 (5)
N1	0.047 (4)	0.042 (4)	0.025 (4)	0.013 (3)	0.000 (3)	−0.008 (3)
N2	0.045 (4)	0.026 (4)	0.027 (3)	0.006 (3)	0.010 (3)	0.002 (3)
N3	0.052 (5)	0.040 (4)	0.038 (4)	0.006 (3)	0.005 (4)	0.004 (3)
N4	0.058 (5)	0.034 (4)	0.031 (4)	0.011 (3)	0.019 (4)	−0.004 (3)
N5	0.053 (5)	0.034 (4)	0.035 (4)	0.009 (3)	0.007 (4)	−0.002 (3)
C1	0.033 (5)	0.030 (4)	0.029 (4)	0.002 (4)	−0.005 (4)	−0.001 (3)
C2	0.045 (5)	0.032 (5)	0.033 (5)	−0.003 (4)	0.005 (4)	0.001 (4)
C3	0.068 (7)	0.034 (5)	0.054 (6)	0.007 (4)	0.025 (5)	−0.001 (4)
C4	0.072 (7)	0.039 (5)	0.038 (5)	0.020 (5)	0.012 (5)	−0.001 (4)
C5	0.043 (5)	0.043 (5)	0.040 (5)	−0.009 (4)	0.014 (4)	−0.015 (4)
C6	0.059 (6)	0.072 (7)	0.040 (5)	−0.005 (5)	0.018 (5)	−0.006 (5)
C7	0.059 (6)	0.074 (8)	0.029 (5)	−0.019 (5)	0.018 (5)	−0.010 (5)
C8	0.043 (6)	0.082 (8)	0.043 (6)	0.004 (5)	0.008 (5)	0.019 (5)
C9	0.051 (6)	0.052 (6)	0.026 (4)	−0.008 (4)	0.009 (4)	0.007 (4)
C10	0.037 (5)	0.049 (5)	0.027 (4)	0.001 (4)	0.001 (4)	−0.004 (4)
C11	0.062 (6)	0.035 (5)	0.048 (6)	0.019 (4)	0.013 (5)	0.007 (4)
C12	0.056 (6)	0.049 (5)	0.030 (5)	0.031 (5)	−0.009 (4)	−0.009 (4)
C13	0.080 (7)	0.030 (5)	0.037 (5)	0.005 (4)	0.024 (5)	−0.005 (4)
C14	0.044 (5)	0.021 (4)	0.034 (4)	0.000 (3)	−0.001 (4)	−0.004 (3)
C15	0.052 (6)	0.037 (5)	0.040 (5)	−0.004 (4)	0.019 (4)	−0.007 (4)
C16	0.042 (5)	0.036 (5)	0.054 (6)	−0.011 (4)	0.010 (4)	−0.010 (4)
C17	0.062 (6)	0.024 (4)	0.057 (6)	−0.005 (4)	0.026 (5)	−0.006 (4)
C18	0.050 (5)	0.023 (4)	0.038 (5)	0.006 (4)	0.015 (4)	0.002 (4)
C19	0.048 (6)	0.044 (5)	0.028 (5)	0.006 (4)	0.012 (4)	−0.008 (4)
C20	0.056 (7)	0.104 (10)	0.104 (9)	−0.015 (7)	0.040 (7)	−0.002 (8)
C22	0.141 (14)	0.199 (19)	0.132 (13)	−0.042 (13)	0.085 (13)	−0.032 (13)

### Geometric parameters (Å, º)

**Table:**

Ni1—N1	1.997 (6)	C5—C10	1.406 (11)
Ni1—N3	2.044 (7)	C5—C6	1.450 (11)
Ni1—O1	2.049 (5)	C6—C7	1.337 (12)
Ni1—N4	2.064 (7)	C6—H6	0.9300
Ni1—O2	2.128 (7)	C7—C8	1.369 (12)
Ni1—N2	2.241 (6)	C7—H7	0.9300
S1—C18	1.641 (9)	C8—C9	1.396 (11)
S2—C19	1.673 (10)	C8—H8	0.9300
O1—C2	1.344 (9)	C9—C10	1.438 (11)
O2—C20	1.411 (11)	C9—H9	0.9300
O2—H2	0.818 (10)	C11—H11	0.9300
O3—C22	1.339 (13)	C12—C13	1.521 (12)
O3—H3	0.8200	C12—H12A	0.9700
N1—C11	1.281 (10)	C12—H12B	0.9700
N1—C12	1.468 (9)	C13—H13A	0.9700
N2—C13	1.487 (9)	C13—H13B	0.9700
N2—C14	1.495 (9)	C14—C15	1.546 (10)
N2—C17	1.505 (10)	C14—H14A	0.9700
N3—C18	1.146 (9)	C14—H14B	0.9700
N4—C19	1.128 (9)	C15—H15A	0.9700
N5—C16	1.473 (9)	C15—H15B	0.9700
N5—C15	1.488 (10)	C16—C17	1.525 (10)
N5—H5A	0.9000	C16—H16A	0.9700
N5—H5B	0.9000	C16—H16B	0.9700
C1—C2	1.403 (10)	C17—H17A	0.9700
C1—C10	1.454 (10)	C17—H17B	0.9700
C1—C11	1.455 (11)	C20—H20A	0.9600
C2—C3	1.390 (11)	C20—H20B	0.9600
C3—C4	1.386 (12)	C20—H20C	0.9600
C3—H3A	0.9300	C22—H22A	0.9600
C4—C5	1.402 (11)	C22—H22B	0.9600
C4—H4	0.9300	C22—H22C	0.9600
			
N1—Ni1—N3	172.3 (3)	C9—C8—H8	119.1
N1—Ni1—O1	87.7 (2)	C8—C9—C10	120.4 (8)
N3—Ni1—O1	96.3 (2)	C8—C9—H9	119.8
N1—Ni1—N4	91.9 (3)	C10—C9—H9	119.8
N3—Ni1—N4	94.6 (3)	C5—C10—C9	118.0 (7)
O1—Ni1—N4	91.0 (2)	C5—C10—C1	119.7 (7)
N1—Ni1—O2	88.8 (3)	C9—C10—C1	122.3 (7)
N3—Ni1—O2	84.9 (3)	N1—C11—C1	125.3 (8)
O1—Ni1—O2	86.6 (2)	N1—C11—H11	117.4
N4—Ni1—O2	177.4 (2)	C1—C11—H11	117.4
N1—Ni1—N2	81.9 (2)	N1—C12—C13	106.7 (7)
N3—Ni1—N2	93.8 (2)	N1—C12—H12A	110.4
O1—Ni1—N2	169.1 (2)	C13—C12—H12A	110.4
N4—Ni1—N2	92.3 (2)	N1—C12—H12B	110.4
O2—Ni1—N2	90.3 (2)	C13—C12—H12B	110.4
C2—O1—Ni1	123.5 (4)	H12A—C12—H12B	108.6
C20—O2—Ni1	126.9 (6)	N2—C13—C12	110.2 (7)
C20—O2—H2	116 (8)	N2—C13—H13A	109.6
Ni1—O2—H2	117 (7)	C12—C13—H13A	109.6
C22—O3—H3	109.5	N2—C13—H13B	109.6
C11—N1—C12	118.5 (7)	C12—C13—H13B	109.6
C11—N1—Ni1	127.3 (6)	H13A—C13—H13B	108.1
C12—N1—Ni1	113.8 (5)	N2—C14—C15	113.6 (6)
C13—N2—C14	112.6 (6)	N2—C14—H14A	108.9
C13—N2—C17	112.5 (6)	C15—C14—H14A	108.9
C14—N2—C17	107.1 (6)	N2—C14—H14B	108.9
C13—N2—Ni1	102.8 (4)	C15—C14—H14B	108.9
C14—N2—Ni1	112.8 (4)	H14A—C14—H14B	107.7
C17—N2—Ni1	109.0 (4)	N5—C15—C14	110.6 (6)
C18—N3—Ni1	159.3 (7)	N5—C15—H15A	109.5
C19—N4—Ni1	171.1 (7)	C14—C15—H15A	109.5
C16—N5—C15	110.0 (6)	N5—C15—H15B	109.5
C16—N5—H5A	109.7	C14—C15—H15B	109.5
C15—N5—H5A	109.7	H15A—C15—H15B	108.1
C16—N5—H5B	109.7	N5—C16—C17	111.0 (7)
C15—N5—H5B	109.7	N5—C16—H16A	109.4
H5A—N5—H5B	108.2	C17—C16—H16A	109.4
C2—C1—C10	118.0 (7)	N5—C16—H16B	109.4
C2—C1—C11	123.1 (7)	C17—C16—H16B	109.4
C10—C1—C11	118.8 (7)	H16A—C16—H16B	108.0
O1—C2—C3	115.9 (7)	N2—C17—C16	113.3 (6)
O1—C2—C1	123.2 (7)	N2—C17—H17A	108.9
C3—C2—C1	120.9 (7)	C16—C17—H17A	108.9
C4—C3—C2	121.1 (8)	N2—C17—H17B	108.9
C4—C3—H3A	119.4	C16—C17—H17B	108.9
C2—C3—H3A	119.4	H17A—C17—H17B	107.7
C3—C4—C5	120.2 (8)	N3—C18—S1	177.8 (8)
C3—C4—H4	119.9	N4—C19—S2	176.6 (8)
C5—C4—H4	119.9	O2—C20—H20A	109.5
C4—C5—C10	120.0 (7)	O2—C20—H20B	109.5
C4—C5—C6	122.8 (8)	H20A—C20—H20B	109.5
C10—C5—C6	117.2 (8)	O2—C20—H20C	109.5
C7—C6—C5	124.1 (9)	H20A—C20—H20C	109.5
C7—C6—H6	118.0	H20B—C20—H20C	109.5
C5—C6—H6	118.0	O3—C22—H22A	109.5
C6—C7—C8	118.6 (8)	O3—C22—H22B	109.5
C6—C7—H7	120.7	H22A—C22—H22B	109.5
C8—C7—H7	120.7	O3—C22—H22C	109.5
C7—C8—C9	121.8 (9)	H22A—C22—H22C	109.5
C7—C8—H8	119.1	H22B—C22—H22C	109.5

### Hydrogen-bond geometry (Å, º)

**Table:**

*D*—H···*A*	*D*—H	H···*A*	*D*···*A*	*D*—H···*A*
O2—H2···O3	0.82 (1)	2.03 (4)	2.793 (10)	155 (10)
N5—H5*B*···O1^i^	0.90	1.75	2.649 (8)	175
N5—H5*A*···S2^ii^	0.90	2.67	3.480 (7)	150
O3—H3···S2^ii^	0.82	2.78	3.532 (9)	154

Symmetry codes: (i) −*x*, *y*−1/2, −*z*+1/2; (ii) *x*, −*y*+1/2, *z*−1/2.
